# Influence of Crossrib Configuration on Bond-Slip Behavior for High-Strength Reinforcement in Concrete

**DOI:** 10.3390/ma18143221

**Published:** 2025-07-08

**Authors:** Sisi Chao

**Affiliations:** 1 School of Architecture Engineering, Xi’an Technological University, No. 2 Xuefuzhonglu Road, Weiyang District, Xi’an 710021, China; chaosisi@xatu.edu.cn; Tel.: +86-18291820106; 2 Xi’an Key Laboratory of Urban Low-Carbon Construction, Chang’an University, Xi’an 710061, China

**Keywords:** high-strength steel rebar, bond behavior, rib outline, critical bond length, bond-slip constitutive model

## Abstract

In the present study, the mechanical properties of high-strength steel rebar with different crossrib spacing that affect the bond behavior between steel rebar and concrete is investigated. To reveal the effects of crossrib spacing on the bond behavior of 630 MPa high-strength steel rebar (T63) in concrete, 42 bonding specimens were designed using T63 rebars and T63 rebars with increased crossrib spacing (TB63). The bond properties of two kinds of steel rebar with concrete were investigated by pull-out test and the failure modes, bond strengths, relative slippages, and bond-slip curves were obtained. Based on analysis of bond-slip curves, the applicability of the existing bond-slip constitutive model to describe T63 and TB63 rebars was discussed. It was found that 30–50% increase in crossrib spacing had little effect on the bond failure mode and bond strength of T63 rebar. The bond-slip curves of the two types of bonding specimens were similar and there is a 1.3 to 1.5-fold increase in peak slippage with TB63. The calculation method of critical bond length in Chinese code (GB 50010-2010) is applicable to T63 and TB63 rebars, and the bond-slip characteristics of T63 rebar with different crossrib spacings was reliably described by the bond-slip constitutive model. The research results can be used as the basis for the application of T63 reinforcement and can also be used as a reference for optimizing of rebar ribs outline.

## 1. Introduction

In transportation infrastructure’s lifecycle, persistent high-frequency fatigue loading drives traditional low-strength reinforcement into dual dilemmas: excessive density causes concrete compaction defects, while bond-slip degradation accelerates structural deterioration. This compels high-strength reinforcement to emerge as a critical solution, whose interfacial bond mechanics directly govern load transfer efficiency in steel–concrete systems. Clark et al. [1,2] pioneered the systematic investigation of reinforcement geometric parameters’ influence on bond performance, establishing fundamental principles for rib geometry design through extensive experimental studies. The main differences between the rebars used were the shape and size of the surface configuration, including rib spacing and rib height. Based on experimental studies, the following recommendations for steel rebar rib geometries was proposed. The shearing area of steel rebars (i.e., the perimeter of the cross-section of steel bars multiplied by the rib spacing) and the rib bearing area (i.e., the projected area of rib perpendicular to rebar axis) should not exceed 10, with 5–6 appropriate. Based on Clark’s research, the American Standard ASTM A305-49 [3] has been amended regarding the rebar surface configuration to specify that the spacing between rebar crossribs is not greater than 0.7 times the rebar nominal diameter. The minimum values of the crossrib height of the rebar with different diameters are 4, 4.5, and 5% rebar nominal diameter (corresponding to rebar with a diameter <13, 13–16, and >16 mm, respectively). This proposal is still in use in many nations. With the widespread application of high-strength reinforcement in engineering practices, the bond performance between reinforcement and concrete has become a critical concern. Currently, the geometric parameters of reinforcement bars are primarily based on research findings from traditional reinforcement. Recent research by Shunmuga Vemb et al. [4] further validated these basic conclusions in modern high-strength concrete, highlighting the particularly significant impact of rib height-to-spacing ratio on bond strength. Yang et al. [5] conducted a systematic comparison of bond mechanism interpretations across different design codes, noting that, while specific parameter limits vary among national standards, they universally recognize mechanical interlocking as the primary mechanism for bond between high-strength reinforcement and concrete. However, whether these findings are applicable to new-generation reinforcement with significantly enhanced mechanical properties requires further investigation.

Bond anchorage between reinforcement and concrete is one of the fundamental issues in reinforced concrete structures, characterized by complex mechanisms and numerous influencing factors [6,7]. Therefore, the bond behavior between the two materials has been a difficult problem to study in reinforced concrete structures [8,9]. Lutz et al. [10] identified two primary mechanisms of relative slip between deformed bars and concrete: the wedging action of concrete between ribs and crushing of concrete in front of ribs. When the rib face angle exceeds 40°, slip mainly occurs due to concrete crushing in front of ribs; when the angle is less than 30°, bond performance significantly decreases due to reduced friction. Skorobogatov et al. [11] supported these findings through comparative studies of bars with 48.7° and 57.8° rib angles. Esfahani et al. [12] further confirmed that bars with rib angles of 23–27° exhibited inferior bond performance compared to those with 40–47° angles. Soretz [10] investigated the effects of various rib parameters, including height, spacing, angle, and shape, finding that proportional changes in rib spacing and height had minimal impact during small slip stages (<1 mm). However, when slip exceeded 1 mm, the bond strength of bars with the smallest rib height decreased by 20% compared to other bars. Soretz [13] recommended rib spacing and height to be 0.3 and 0.03 times the bar diameter respectively, and found that rib angles between 40° and 70° had no significant effect on bond strength. Darwin et al. [14,15,16] conducted comprehensive beam tests revealing that bond-slip characteristics result from the combined effect of multiple geometric parameters. They found that increasing relative rib area enhanced initial stiffness, leading to the development of calculation formulas incorporating relative rib area. The formulae for calculating bond strength and bond length considering the relative rib area of the steel rebars have been established based on this testing. Xiong [17] conducted pullout tests on 21 high-strength bars with special spiral grooves to study various parameters’ effects on bond behavior. The study revealed that increased cover thickness improved bond strength, and bars with six spiral grooves showed better performance than those with three grooves. Adding stirrups changed failure modes from splitting to splitting-pullout failure. Liu [18] developed an analytical solution for the reinforcement-UHPC bond-slip relationship, considering fiber bond properties at fine scale. The study provided critical point coordinates for different stages and presented simplified equations using thick-walled cylinder theory, virtual crack model, and fiber-matrix discrete model, offering a new approach to study UHPC-deformed bar bonding properties. Zhang [19] examined bonding properties of helically grooved ultra-high-strength steel bars with high-strength recycled concrete through pullout tests. Variables included concrete strength (50–60 MPa), recycled concrete aggregate (RCA) replacement rates (0%, 50%, and 100%), embedment length (5 d, 10 d, and 20 d), cover protection layer (25 mm, 45 mm, and 68.7 mm), and other parameters. An empirical bond stress–slip relationship model was developed, showing good agreement with experimental results and providing guidance for UHSSB applications. Recent studies have employed various testing methods to evaluate bond behavior between concrete and reinforcement. For instance, Shafaie et al. [20] utilized slant shear tests combined with fuzzy logic to assess bond strength in concrete repair applications, demonstrating the complexity and importance of bond strength evaluation in concrete structures.

The evolution of deformed steel bars in China since the 1950s has progressed from Soviet-introduced bamboo steel rebar through herringbone and spiral types, eventually settling on crescent rib steel rebars as the standard choice after finding limitations in high rib steel bars. Xu’s pull-out test research [21] revealed important comparative performance characteristics among different bar types. While high-ribbed bars demonstrated superior initial bond strength and stiffness compared to crescent-ribbed bars, their concrete “meshing gear” was susceptible to crushing, resulting in rapid strength deterioration. Steel stranded and torsional bars exhibited interesting dual characteristics, behaving like plain bars in the pre-stress stage but similar to deformed bars during large slips. Notably, spiral ribbed steel bars emerged as a particularly effective option, combining the advantages of both torsional and ribbed bars with high bond strength and minimal slip. Meanwhile, studies on shape parameters [22,23] provided crucial insights into design considerations. Research showed that, while increasing rib height enhanced bonding performance, it compromised the bars’ mechanical properties. Similarly, decreased rib spacing improved interlocking effects but led to reduced shear resistance. These findings led to the adoption of relative rib area as a key parameter for evaluating surface configuration performance. As there are many restrictions on the surface configuration of deformed steel bars, the manufacturing of steel bars and their use in concrete comply with these standard restrictions and, thus, research on variable surface parameters of steel bars in China is almost a blank.

After entering the new century, both the mechanical properties of steel bars and the shape characteristics of steel bars have been further optimized [24,25,26]. At present, crescent ribbed bars are the only deformed reinforcement used in concrete structures in China [27], mainly following the relevant research results of 30 years ago, and whether these research findings are still applicable to the new type of steel bars with substantially improved mechanical properties requires further study. Although extensive research has been conducted on conventional reinforcing bars, there remains a significant knowledge gap in the geometric optimization of high-strength reinforcement. The bond mechanisms and existing geometric parameter design criteria, primarily developed for normal-strength reinforcement, need to be re-evaluated for high-strength bars due to their distinct material properties and surface characteristics. Furthermore, there is a pressing need to optimize the geometric configuration of high-strength reinforcement to balance bond performance and manufacturing efficiency. To address these issues, this study investigated the bond behavior through comparative pull-out tests of crescent ribbed T63 bars with standard and increased rib spacing. Analysis of bond strength, bond-slip characteristics, and failure modes from 42 pull-out specimens provides fundamental insights into the optimization of high-strength reinforcement geometry and establishes reference data for their manufacture and application.

## 2. Test Procedure

### 2.1. Materials

#### 2.1.1. Surface Configuration of Rebar

**Figure 1:**
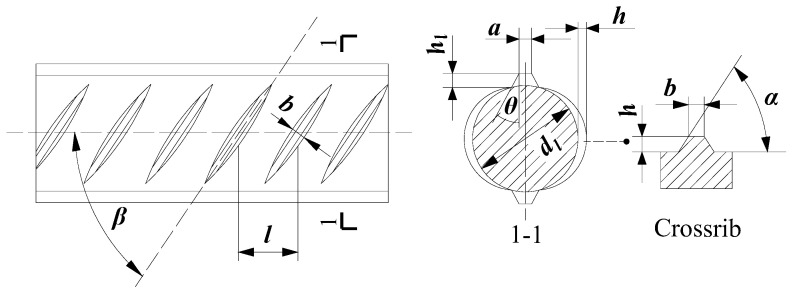
Surface configuration of rebar. *d*_1_—internal diameter; *α*—crossrib inclination angle; *h*—crossrib height; *β*—crossrib angle with axis; *h*_1_—longitudinal rib height; *θ*—longitudinal rib inclination angle; *a*—longitudinal rib top width; *l*—crossrib spacing; *b*—crossrib top width.

Hot-rolled ribbed steel bars are the main steel bars used in concrete structures today, with longitudinal ribs parallel to the axis of the bar and crossribs at a certain inclination to the axis (Figure 1). The steel bars used in the present tests were high-strength rebar with a yield strength of 630 MPa produced by a manufacturer in Jiangsu, China. Two types of high-strength rebar were used for the bonding performance test, one was the standard high-strength rebar (T63) following the Chinese Code (GB/T 1499.2-2018) [27], and the other was T63 rebar with increased crossrib spacing (termed here as TB63). These two types of rebar have the same surface parameters except for the different crossrib spacing and include three diameters, 16, 22, and 25 mm. The production of crescent pattern rebar with different profile characteristics involves the manufacturing of rebar forming moulds, which is limited by the manufacturing process of rebar molds. In this paper, the selection of increased rib spacing was based on several key considerations. Firstly, the current standard GB/T 1499.2-2018 specifies allowable deviations in rib spacing of ±0.5 to 0.8 mm, which provided a baseline reference. Additionally, manufacturing feasibility was take into account, indicating that the increase in spacing should remain within practical limits. Therefore, the variation of the cross rib spacing of the rebar is used to locate 38%, 31.8%, and 54.7%, respectively. Before tests, 6 steel rebars of different diameters were randomly selected for measuring crossrib spacing (Table 1).

#### 2.1.2. Mechanical Properties of Reinforcing Steel

Tensile testing of the steel bars was conducted according to the Chinese Code (GB/T 228.1-2021) [28]. As the rebar crossribs were not involved in the force when the steel rebars were in tension, only T63 rebars were tested (Table 2).

#### 2.1.3. Mechanical Properties of Concrete

Concrete with strength grades of C30, C40, and C50 were selected to prepare bonding specimens. Concrete compressive strength specimens were prepared according to the Chinese Code (GB 50081-2002) [29] and cured under the same conditions as bonding specimens (20 ± 3 °C and relative humidity, RH, 60 ± 10%) for 28d. The measured strengths were 40.69 MPa, 50.32 MPa, and 56.02 MPa for C30, C40, and C50 concrete, respectively. The concrete mix and its mechanical properties are shown in Table 3.

### 2.2. Design and Preparation of Bonding Specimen

**Figure 2:**
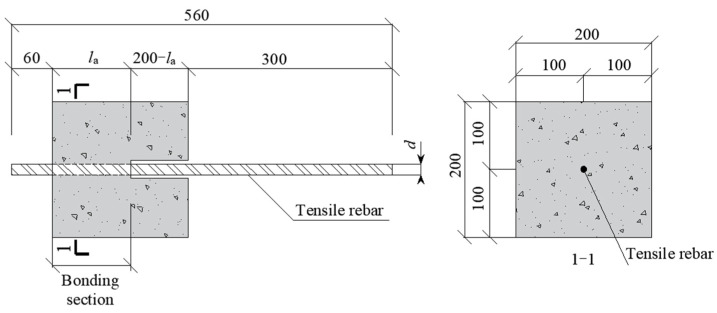
Pull-out test specimen.

The pull-out test method [30,31,32] is the most widely used bonding test method because of the simplicity of specimen production, convenience of loading, and ease of measuring test data, especially in its sensitivity to changes in the surface configuration of steel rebar. In this study, bond testing was conducted using the pull-out specimen shown in Figure 2.

**Figure 3:**
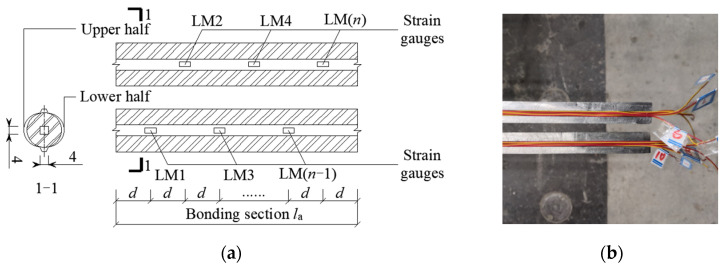
Location distribution of steel rebar strain gauge: (**a**) Strain gauge distribution; (**b**) Strain gauge wire.

The substantial part of the specimen was shaped as a prism, with the steel rebars running through the concrete from the middle and the loaded end of the steel rebars decoupled from the surrounding concrete through a plastic sleeve without hoops. To avoid splitting failure, the concrete cross-section size was chosen as 200 × 200 mm. The rebars at the free end protruded 60 mm from the concrete, and rebar length at the loaded end ~300 mm, according to the requirements of the test device. To measure the stress and strain data of the bonded section without destroying the rebar surface, rebars were slotted along the longitudinal ribs and rewelded together after applying strain gauges inside the bonded section with concrete. To monitor the local strain development during testing, the strain measurements were conducted using BX120-5AA electrical resistance strain gauges with a gauge factor of 2.08 ± 1%, resistance of 120.0 ± 0.1 Ω, and transverse sensitivity coefficient of 0.1%. The welded joints were then polished back to their original state. For different bond lengths (5*d*, 7*d* and 10*d*), 4, 6, and 9 strain gauges were crossed in the steel rebars bond area (Figure 3).

Three specimens were prepared for each group, for 42 specimens in total (Table 4) and all specimens placed in a natural environment for curing (20 ± 3 °C, RH 60 ± 10%). Specimen surfaces were covered with a wet cotton cloth to prevent them from losing water too quickly and producing dry cracks. Specimens were demolded after 1d and their surfaces again covered with wet cotton cloth after demolding for daily watering and curing for 13d, followed by natural curing in a rain shelter for 14d before testing.

### 2.3. Loading and Measuring

The pull-out test is loaded by a tensile and compression testing machine (maximum range 500 kN) and a steel fixture. The loading system was as follows: (a) pre-loading stage, 3 kN of tensile force applied to the specimen to ensure close contact between the specimen and counterforce frame; (b) loading stage, force-controlled loading method used first, with a loading rate of 6 kN/min, until 60% of the estimated peak load; and (c) then replaced to the displacement-controlled loading method, with a loading rate 0.5 mm/min until rebar debonding or concrete failure. This is to prevent the force-controlled loading method from breaking suddenly and not being able to obtain the descending section of the bond-slip curve.

**Figure 4:**
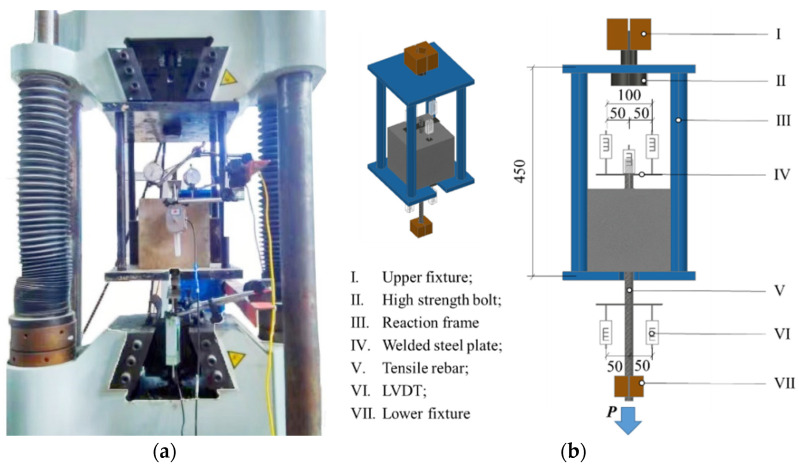
Loading device and measurement points: (**a**) Loading device; (**b**) Distribution of displacement measuring points.

The rebar strain and relative slip were measured using a high-speed static strain test and analytical system with linear variable differential transformers and embedded strain gauges. The test loading device and measurement point arrangement are shown in Figure 4.

## 3. Discussion of Test Results

### 3.1. Failure Modes

**Figure 5:**
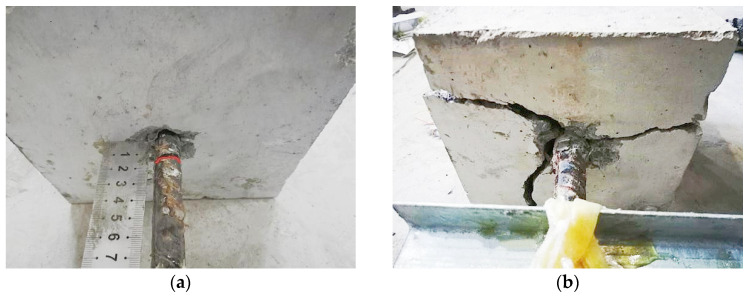
Typical failure mode of bonding specimens: (**a**) Debonding; (**b**) Concrete splitting.

The failure modes of the bonding specimens showed that debonding failure occurred in specimens with short bond lengths (5*d* and 7*d*) or low concrete strength (C30, Table 5, Figure 5a). Such specimens showed rapid load growth at the beginning of loading and slippage began to occur at both the free and loaded ends near peak load. After the load dropped to 30% of peak load, the rebars slowly pulled out. The concrete splitting failure mode occurred in specimens with long bond lengths (10*d*) or diameters of rebars as large as 22 mm and 25 mm (Figure 5b). For this kind of specimen, there was no clear phenomenon before the load reached its peak and there was no slip at the free and loading ends. After the load reached the peak, the concrete part of the specimen suddenly split into 2 or 3 pieces, with the split concrete surfaces left with marks of the ribs and concrete powder attached at front of the ribs.

### 3.2. Bond Strength

Based on peak loads collected in these tests, the peak bond strength is calculated according to the Equation (1)

(1) $$\tau_{u}=\frac{F_{u}}{\pi dl_{a}}$$

where *τ*_u_ is the bond strength (MPa), *F*_u_ is the maximum measured pullout force (N), *d* is the nominal diameter of the reinforcing bar (mm), and *l*_a_ the bond length (mm). The bond strength was the average value of three specimens (Table 6).

The following is a comparative analysis of the bonding performance of T63 and TB63 specimens from concrete strength, rebar diameter, and bond length, where the rebar diameter was converted to the relative protection layer thickness.

**Figure 6:**
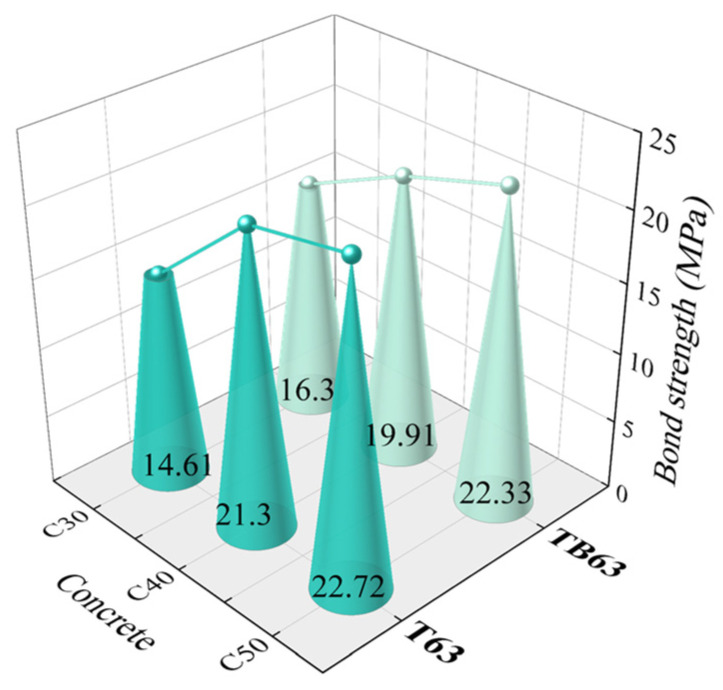
Effects of concrete strength.

(a) Concrete strength. Test results from specimens with a bond length of 7*d* and concrete strengths of C30, C40, and C50 were plotted (Figure 6) and the bond strength of both T63 and TB63 rebar to concrete observed to increase with concrete strength. For T63 specimens, the bond strength increase was greater when the concrete design strength (CDS) grade increased from C30 to C40, while only a slight increase in bond strength occurred when the CDS increased from C40 to C50. This was mainly because, when the CDS reached C40 and C50 in the test, T63 specimens exhibited a substantial splitting failure. Such failure mode did not fully display the bonded bearing capacity of the rebar and concrete.

The conclusion was evident that the bond strength of steel rebars and concrete increased with increased CDS, mainly due to increased cementation and mechanical interactions between rebar and concrete. As CDS increased, the load required to break the concrete in front of the ribs increased and concrete between the ribs more difficult to experience shear failure. At the same time, with increased concrete tensile strength, the circumferential tensile force required to produce internal cracks in a specimen also increased and internal cracks would develop later and expand more slowly. When the CDS increased to a certain critical value, the rebar might break under tensile force.

**Figure 7:**
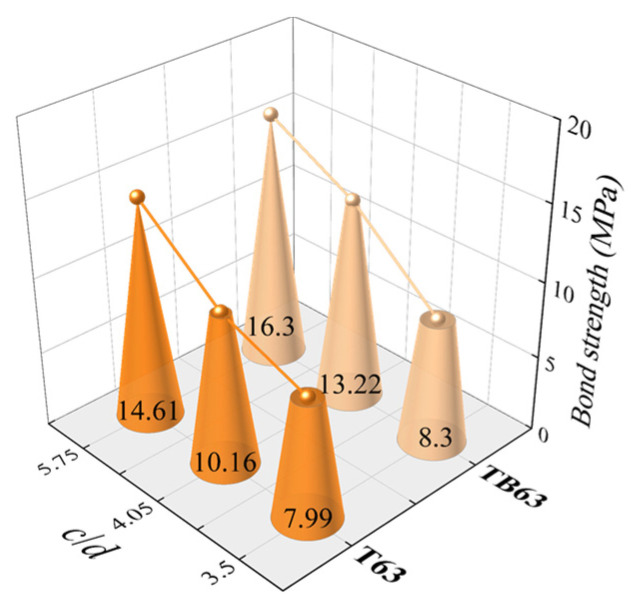
Effects of rebar diameter.

(b) Relative protection layer thickness. For specimens with C30 CDS, 7*d* bond length and 16, 22 and 25 mm rebar diameter, the relative protection layer thicknesses *c*/*d* were 5.75, 4.05, 3.50, respectively, and the corresponding bond strength calculated (Figure 7). With increased relative protection layer thickness, the bond strength of the two types of steel rebars increased. This effect of the protection layer thickness on bond strength was approximately explained according to the LAME solution of thick-walled cylinders in elastic mechanics. For a thick-walled cylinder subjected to a specific internal pressure, the circumferential tensile force can be calculated according to the Equation (2) [33].

(2) $$\sigma =\frac{a^{2}q}{b^{2}-a^{2}}\left(\frac{b^{2}}{r^{2}}+1\right)$$

where *σ* is the circumferential tensile force, *q* the radial pressure in the cylinder, *a* and *b* the inner and outer diameters of the cylinder, and *r* the distance from a point on the cylinder to the circle center. The *a* and *b* in Equation (2) were replaced by rebar diameter *d* and protection layer thickness *c*.

(3) $$\sigma =\frac{{\left(d/2\right)}^{2}}{r^{2}}\left[1+\frac{{\left(d/2\right)}^{2}+r^{2}}{{\left(c+d/2\right)}^{2}-{\left(d/2\right)}^{2}}\right]$$

As seen in Equation (3), the greater the protection layer thickness was, the smaller the circumferential tension on the concrete and the more excellent the concrete crack resistance at the rebar periphery, thus improving the concrete splitting strength and bond strength. When the concrete protection layer thickness increased to a critical value, the concrete in front of the rib failed before the internal crack expanded to the surface. The bond strength was thus no longer affected by circumferential tension, such that bond strength no longer increased with increased protection layer thickness.

**Figure 8:**
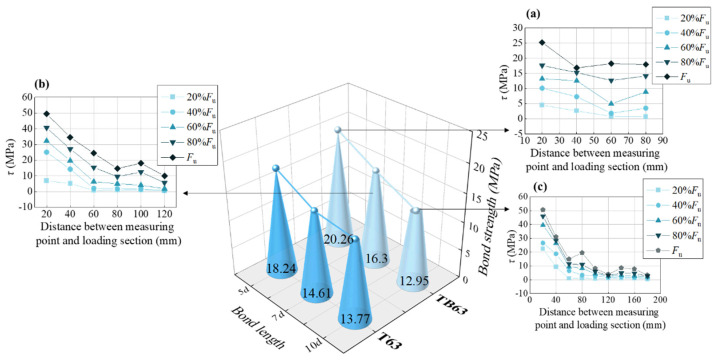
Effects of bond length. (**a**) The bond stress distribution of 5*d* bond length; (**b**) The bond stress distribution of 7*d* bond length; (**c**) The bond stress distribution of 10*d* bond length.

(c) Bond length. The relationships between bond strength and bond length were plotted and the bond strengths of both specimen types observed to generally decrease as bond length increased (Figure 8). Bond length’s effect on bond strength was mainly related to the distribution of bond stresses within the bonding section. The bond stress calculated in the pull-out test, according to Equation (1), was the mean value in the bonding section. In practical terms, the bond stress was not uniformly distributed. When bond length was short, the bond stress distribution was more uniform and the large bond stress section accounted for more of the bonding section (Figure 8a), such that the calculated average bond stress was large. As bond length increased, the bond stress distribution became increasingly uneven (Figure 8b) and the proportion of the large bond stress section small, such that the average bond strength was small (Figure 8c).

**Figure 9:**
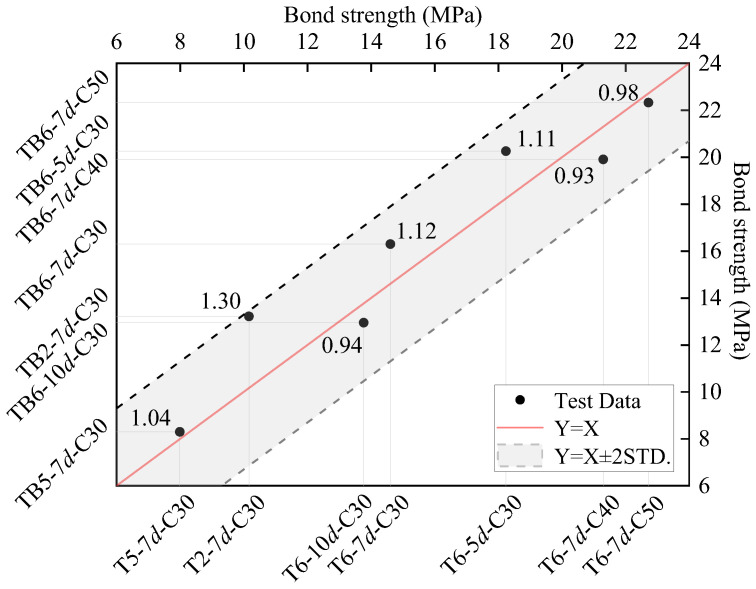
Comparison of average bond strengths of T63 and TB63 specimens. The *x* and *y*-axes, the average bond strength of T63 and TB63 bond specimens, respectively.

Figure 9 shows the comparison of average bond strengths between T63 and TB63 specimens. The red solid line represents the ideal 1:1 correlation (Y = X), while the dashed lines indicate the ±2 standard deviation bounds. Most test data points fall within this range and cluster near the 1:1 line, demonstrating good correlation in bond performance between the two rebar types. The relatively small scatter of data points indicates high reliability of the test results. Overall, the bond strength values for both T63 and TB63 specimens range from 8 to 22 MPa, suggesting that both rebar types provide adequate bond strength.

### 3.3. Bond-Slip Curve

**Figure 10:**
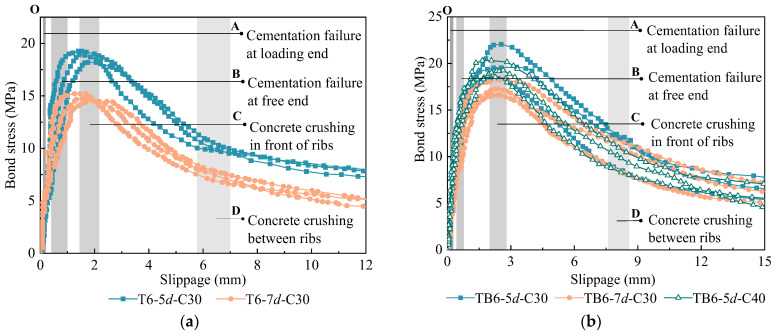
Bond-slip curves: (**a**) T63 specimens; (**b**) TB63 specimens.

The bond-slip curves for specimens with concrete splitting failure could not be measured because of the small amount of slip of the rebars and the fact that the damage occurred within a short period. Accordingly, the bond-slip curves of specimens with debonding failure were analyzed below, with the curves of various types of specimens divided into five stages (Figure 10).

(a) Elastic stage: During the initial loading period, the bond force was mainly provided by cementation between the rebar and concrete at the loading end. As the load increased, the bond force was gradually transferred to the free end until the cementation at the loading end failed. As the bond force at this stage was mainly provided by cementation, there was no significant slip (Figure 10, OA stage).

(b) Initial slip stage: When cementation at the loading end failed, the bonding stress at the loading end was provided by mechanical interactions and friction resistance. With the load increased, the rebar crossribs compressed the concrete in front of the rib and began to slip at the loading end. Subsequently, cementation in the rebar bonding section was successively lost. Finally, this stage ended with failure of cementation at the free end. The slippage in this stage was small and the bond-slip curve rose linearly (Figure 10, A–B stage).

(c) Peak stage: With increased load, the pressing force of the rebar at the crossrib front gradually increased, such that the concrete in front of the crossrib was gradually crushed and the concrete around the crossrib began to develop small internal cracks. Thus, bond stiffness was reduced and the bond-slip curve showed an upward bend. Therefore, bond stiffness decreased and the bond-slip curve presented an upward trend (Figure 10, B–C stage).

(d) Drop stage: After the load reached peak value, concrete in front of the crossrib had been destroyed, bond stress began to drop, and large slip occurred. With increased slip, the concrete failure zone between ribs continuously expanded, the bond force significantly decreased, and the bond-slip curve dropped rapidly. When the concrete between ribs had failed, this stage ended (Figure 10, C–D stage).

(e) Residual stage: After concrete between the ribs had failed, the mechanical interactions between the crossrib and concrete were lost and the bond force thereafter mainly caused by friction resistance. Therefore, the bond-slip curve was basically in a horizontal state and, finally, the rebar slowly pulled out (Figure 10, after D stage).

The shapes of bond-slip curves of the two types of rebar and concrete were observed to be the same (Figure 10). The research results indicate that the TB63 reinforcement with a 30% increase in rib spacing performs similarly to the standard T63 reinforcement in terms of bond performance. As shown in Table 7, the bond strength (*τ*_u_) ranges from 14.61 to 20.26 MPa across different specimens, with peak slip values (*S*_u_) between 1.67 and 2.52 mm. The TB6-5*d*-C30 specimen achieved the highest bond strength of 20.26 MPa, while the T6-7*d*-C30 showed the lowest at 14.61 MPa. The difference in average bond strength between the two types of reinforcement specimens is less than twice the standard deviation of bond strength, and their bond strength ratio is close to 1, indicating that the increase in rib spacing has minimal effect on bond strength. In terms of bond-slip behavior, both types of reinforcement exhibited similar curve patterns. The peak slip value of TB63 specimens was approximately 1.3–1.5 times that of T63 specimens, with both types reaching peak slippage around 2 mm. When the slippage reached 10 mm and 13.8 mm (corresponding to the rib spacing of T63 and TB63 respectively), the residual bond stress *τ*_r1_ and *τ*_r2_ decreased to approximately 40% and 35% of the bond strength for both types, as indicated by the *τ*_r1_/*τ*_u_ and *τ*_r2_/*τ*_u_ ratios in Table 7. These findings demonstrate that appropriately increasing rib spacing is feasible while maintaining basic bond performance, providing an important basis for optimizing reinforcement geometry design.

## 4. Critical Bond Length

The critical bonding state is when the yielding of steel in tension coincides with the bond stress between the steel rebars and concrete to reach the bond strength. The length at which bond failure occurs is defined as the critical bond length. According to equilibrium conditions, this state corresponds to the limit equilibrium, as expressed in simplification in Equation (4), allowing the critical bond length to be calculated using

(4) $$l_{a}=\frac{f_{y}}{4\tau_{u}}d$$

where *τ*_u_ is the bond strength. This study adopted Equation (5) [34] to calculate the critical bond length. Theoretically, the critical bond length can be obtained by substituting Equation (4) into Equation (5), from which the parameters in Equation (5) were analyzed below.

(5) $$\tau_{u}=\tau_{c}\frac{1+1/M}{1.85+0.024\sqrt{M}}\times \underset{\mathrm{Rebar}\;\mathrm{positioning}\;\mathrm{effect}}{\underset{︸}{\left(0.88+0.12\frac{c_{\mathrm{med}}}{c}\right)}}\times \underset{\mathrm{Stirrup}\;\mathrm{effect}}{\underset{︸}{\left(1+0.015\frac{A_{s}A_{\mathrm{sv}}}{cs}\right)}}$$

(6) $$\tau_{c}=4.7\frac{c/d+0.5}{c/d+3.6}\sqrt{{f_{c}}^{\prime }}$$

(7) $$M=\cosh(0.0022l_{a}\sqrt{\frac{3{f_{c}}^{\prime }}{d}})$$

where *τ*_c_ is the result of Tesfers’ calculation [35] according to Equation (6); *M* the correction coefficient considering the influence of rebar diameter and bond length, calculated according to Equation (7); *c*_med_ = mean{*c*_x_, *c*_y_, *c*_s_} and *c* = min{*c*_x_, *c*_y_, *c*_s_}, with *c*_x_ and *c*_y_ the thickness of the protective layer on the side and bottom of the specimen, respectively, and *c*_s_ the rebar spacing; *A*_s_ the nominal area of tensile rebar; *A*_sv_ the nominal area of a single stirrup; and *s* the stirrup spacing; *f*_c_’ is the cylindrical compressive strength of concrete.

Substituting Equation (5) into Equation (4) provided

(8) $$l_{a}=\frac{f_{y}}{4\tau_{c}\alpha_{1}\alpha_{2}}d$$

(9) $$\alpha_{1}=\frac{1+1/M}{1.85+0.024\sqrt{M}}$$

(10) $$\alpha_{2}=(0.88+0.12\frac{c_{\mathrm{med}}}{c})(1+0.015\frac{A_{s}A_{\mathrm{sv}}}{cs})$$

The parameter *M* of Equation (9) is related to the bond length *l*_a_ and the direct calculation of Equation (8) requires repeated trial calculations, such that it does not apply to the actual design calculation. Esfahani [34] conducted extensive calculations, substituted Darwin’s experimental results [16] to verify, and finally suggested *α*_1_*α*_2_ = 0.85. Substituting this into Equation (8) obtained

(11) $$l_{a}=\frac{f_{y}}{3.4\tau_{c}}d$$

In combination with common practices in engineering and Chinese Code (GB 50010-2010) [36], assuming c = d and substituting it into Equation (8) and Equation (7) obtained

(12) $$l_{a}=0.192\frac{f_{y}}{\sqrt{{f_{c}}^{\prime }}}d$$

The factor reflecting concrete strength in the above equation was the cylindrical compressive strength ${f_{c}}^{\prime }$ , while the Chinese Code (GB 50010-2010) takes concrete tensile strength *f*_t_ as the strength index of concrete. Consequently, it was necessary to convert ${f_{c}}^{\prime }$ in the above equation into *f*_t_. First, ${f_{c}}^{\prime }$ = 0.76*f*_cu_ was substituted into the above equation and in the conversion according to $f_{t}=0.395{f_{\mathrm{cu}}}^{0.55}$ . The *f*_cu_^0.55^ and *f*_cu_^0.5^ of each concrete grade was calculated and the latter was ~1.25-fold of the former. Therefore, the critical bond length was obtained by substituting *f*_cu_^0.55^ = 1.25 *f*_cu_^0.5^ and $f_{t}=0.395{f_{\mathrm{cu}}}^{0.55}$ into Equation (12), to yield

(13) $$l_{a}=0.103\frac{f_{y}}{f_{t}}d$$

The Chinese Code (GB 50010-2010) stipulates that the critical bond length of ribbed steel bars is calculated according to

(14) $$l_{a}=0.14\frac{f_{y}}{f_{t}}d$$

Comparing the above two critical bond length calculation equations, the calculation result of Equation (14) was found to be ~30% larger than that of Equation (13). Due to the small amount of test data in this study, it was difficult to perform statistical reliability analysis on Equation (13), while Equation (14) has been obtained by performing statistical and reliability analyses on a large amount of test data, which had high safety redundancy. The calculation equations of critical bond lengths for 500 and 600 MPa grade steel rebar were obtained from Refs. [37,38], based on the similar methods above, respectively. Their calculated results were both greater than those from Equation (13). Meanwhile, the results of the reliability analysis of the critical bond length were smaller than those from Equation (14) Therefore, the bond lengths of T63 and TB63 rebar calculated by Equation (14) were considered to have acceptable safety redundancy.

## 5. Bond-Slip Constitutive Model

Due to the complex bond mechanism between steel rebar and concrete, the expression of the bond-slip constitutive relation has differed among scholars and codes in different nations. The main constitutive models include single continuous expression and segmental expression. The bond-slip relationship is typically described using a segmental expression to represent its different stages. In this study, several typical constitutive models were selected for analysis.

Harajli [39] has proposed the expressions for the bond-slip curve corresponding to different failure modes based on experimental analysis. In this study, only the curve expression corresponding to debonding failure was taken and the specific expression used was

(15) $$\tau =\left\{\begin{array}{l} \tau_{u}{\left(\frac{S}{S_{1}}\right)}^{0.3}\;\;\;\;\;\;S\leq S_{1}\\ \tau_{u}\;\;\;\;\;\;\;\;\;S_{1}<S\leq S_{2}\\ \tau_{u}+(\tau u-\tau_{r})\frac{S-S_{2}}{S_{2}-S_{3}}\;\;S_{2}<S\leq S_{3}\\ \tau_{r}\;\;\;\;\;\;\;\;\;S\geq S_{3} \end{array}\right.$$

where, *τ*_u_ is the ultimate bond strength (MPa), taken as 2.75 $\sqrt{{f_{c}}^{\prime }}$ ; *τ*_r_ the residual bond strength (MPa), taken as 0.35*τ*_u_; *S*_1_, *S*_2_, and *S*_3_ the slippages (mm) corresponding to ultimate bond and residual strengths, respectively; and *S*_1_ = 0.15 *l*, *S*_2_ = 0.35 *l*, and *S*_3_ = *l*, where *l* is the crossrib spacing.

The Eurocode CEB-FIP Model Code 1990 [40] (MC90) adopts a constitutive model similar to that of Harajli. The power of the rising section is modified to 0.4 and other parameters also modified according to the constraint condition and bonding state (Table 8). The above two constitutive models are the most widely used constitutive models and close to the present test results. Subsequently, the bond-slip constitutive relation of the new type of rebar or concrete was mostly modified from the above model.

Haskett [41] has made the following correction to the constitutive model in MC90, such that the straight section of the curve’s ultimate bond strength is removed. This is because research on existing data has not found that there is a straight section in the bond-slip curve, but it begins to decrease after reaching the ultimate bond strength. The concept of interfacial fracture energy has been introduced and the area between the bond-slip curve and the *x*-axis defined as interfacial fracture energy. Considering that the bond force of the residual section for the curve is mainly friction force, the residual section is removed to close the curve. According to the MC90 model, the intersection of the falling section of the curve and the *x*-axis is 15 mm, which makes the interface fracture energy determined and the implicit calculation method formed. The constitutive model is expressed as

(16) $$\tau =\left\{\begin{array}{l} \tau_{u}{\left(\frac{S}{S_{u}}\right)}^{0.4}\;\;\;\;S\leq S_{u}\\ \tau_{u}+\frac{S-Su}{S_{u}-S_{\max}}\tau_{u}\;S_{u}\leq S\leq S_{\max} \end{array}\right.$$

where, *τ*_u_ is the ultimate bond strength (MPa), taken as 2.5 $\sqrt{{f_{c}}^{\prime }}$ ; *S*_u_ the slippage corresponding to the ultimate bond strength (mm), taken as 1.5 mm; and *S*_max_ the intersection of the curve and *x*-axis, taken as 15 mm.

Mo [42] has found that the ultimate bond strength of Haskett’s constitutive relationship is lower than the test value and has modified the ultimate bond strength based on retaining the residual section of the curve. The expressions of each section are

(17) $$\tau =\left\{\begin{array}{l} \tau_{u}{\left(\frac{S}{S_{u}}\right)}^{0.35}\;\;\;\;\;\;S\leq S_{u}\\ \tau_{u}+\frac{S-Su}{S_{u}-S_{r}}(\tau_{u}-\tau_{r})\;\;S_{u}\leq S\leq S_{r}\\ \tau_{r}\;\;\;\;\;\;\;\;\;S\geq S_{r} \end{array}\right.$$

where *τ*_u_ is the ultimate bond strength (MPa), taken as 3.5 $\sqrt{{f_{c}}^{\prime }}$ ; *τ*_r_ the residual bond strength (MPa), taken as 0.25*τ*_u_; *S*_u_ and *S*_r_ the slippages corresponding to ultimate bond and residual strengths (mm), taken as *S*_u_ = 1 mm and *S*_r_ = *l*, respectively, where *l* is the crossrib spacing.

Xu [25] has proposed a 5-stage bond-slip constitutive model based on experiments and statistics. The model includes micro-sliding, sliding, splitting, descending, and residual stages. The characteristic points between each stage are the bond strength and corresponding slippage (Table 9). The specific expression of the above constitutive model is given in reference [40].

Wu [43] suggested that, to overcome the discontinuity and smoothness of the existing bond-slip constitutive model, a continuous exponential bond-slip constitutive model be obtained through experimental analysis and mathematical derivation. The model adopts the same expression for the failure and bonding states, expressed as

(18) $$\tau =\frac{\tau_{u}}{\left[e^{-B\ln\left(B/D\right)/\left(B-D\right)}-e^{-D\ln\left(B/D\right)/\left(B-D\right)}\right]}(e^{BS}-e^{DS})$$

There are a total of three parameters in the above equation that need to be calculated according to the actual conditions of each specimen (Table 10).

**Figure 11:**
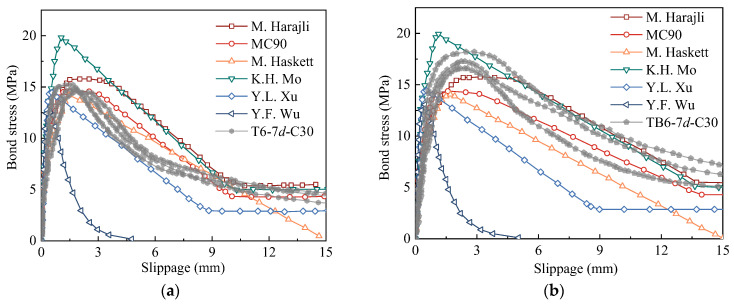
Comparison of test curves and constitutive model curves: (**a**) T6-7d-C30; (**b**) TB6-7d-C30.

To compare and analyze the above constitutive models, T6-7*d*-C30 and TB6-7*d*-C30 test curves and the above constitutive model curves were drawn in the same coordinate (Figure 11). The following observations were obtained from Figure 11:

(a) The segmented constitutive model, except Wu’s constitutive model, was in good agreement with the test curve, which indicated that it was reasonable for describing the bond-slip characteristics of T63 and TB63 rebar and concrete with segmented expression. The constitutive model proposed by Wu was only close to the test curve in the rising section, but its ultimate bond strength and descending section of the curve significantly different from the test curve, especially as the slope change in the descending section was significantly increased compared with the test curve. The factors affecting the two parameters *B* and *D* in the constitutive model were *c*/*d* and *ρ*_sv_ and the latter multiplied by a large coefficient, which had a decisive influence on the shape of the constitutive curve (Table 7). In this study, no stirrup was set in the bonding specimens, such that the test curve was different from the Wu constitutive model curve. Compared with the results of the beam test with stirrups in reference [33], Wu’s constitutive model curve had a better fitting effect. Therefore, the bond-slip constitutive model has been applied to beam tests, but not to pull-out tests, and the applicability of the model for structural analysis considering bond-slip remains to be verified.

(b) Ultimate bond strength is an important factor affecting the bond-slip curve shape. In the constitutive model, ultimate bond strength is often simplified to the quantity only related to concrete strength and the influence of the actual restraint condition and bond length ignored. On the one hand, this was because the concrete strength is the most important factor affecting bond strength and other factors have less influence, especially for debonding failure in the actual structure. On the other hand, this simplified the expression of the constitutive model and facilitated its application. Comparing the ultimate bond stress of the test and constitutive model curves, the bond strengths of Harajli and Mo’s model were found to be close to the test values, while the ultimate bond strengths in other constitutive models were lower than test values. Mo’s model usually slightly overestimates the bond strength of the specimens.

(c) Characteristic slippage has an important influence on the bond-slip curve shape. The peak slippages in the constitutive models of Mo and Xu were small, which led to the slopes of the rising section of the curves, i.e., the bond stiffness, being slightly larger than test results. The peak slippage of MC90 and Haskett’s constitutive model was close to that of T63 rebar but smaller than that of TB63 rebar. The peak slippage of Harajli’s model was close to the test results of the two rebar types. According to the analysis in Section 3.3, the characteristic slippage increased with the crossrib spacing. In Harajli’s constitutive model, each characteristic slippage was a multiple of the crossrib spacing and the effect of the crossrib spacing on the slip considered, which was consistent with the actual situation. In the constitutive models of Harajli, MC90, and Mo, the start of the residual section was when slip reached a crossrib spacing, which was consistent with the test curve. However, the slip of the residual point in Xu’s model was small. In addition, it was still debatable that the intersection of the curve and *x*-axis was set at 15 mm in Haskett’s constitutive model and it is suggested in the reference [43] to set it at twice the crossrib spacing. To make the above constitutive model applicable to rebar with different crossrib spacings, it is suggested here to calculate the characteristic slippage according to the crossrib spacing.

In summary, the constitutive model proposed by Harajli was used for the bond-slip analysis of T63 rebar with different crossrib spacings. If other constitutive models are used, the characteristic slippage and bond strength need to be converted.

## 6. Discussion

Based on the experimental results obtained from this investigation, a comprehensive discussion of the bond performance mechanism of high-strength reinforcement with increased rib spacing is presented herein:

(a) The failure mode transition reveals fundamental interactions between concrete confinement and rib compression effects. For specimens with shorter bond lengths (5d, 7d) or lower strength concrete (C30), the moderate radial pressure enables concrete confinement to effectively resist splitting, manifesting as pullout failure. Conversely, with 10d bond length or larger diameter bars, accumulated radial pressure exceeds confinement capacity, triggering sudden splitting failure. This behavior highlights how geometric and material parameters influence failure mechanisms.

(b) While increased rib spacing (>30% for TB63) minimally impacts bond strength, it significantly affects deformation characteristics. The bond mechanism remains dominated by mechanical interlocking within current design parameters, but TB63 specimens exhibit 30–50% larger peak slip values. This phenomenon stems from the expanded concrete bearing zone between ribs, requiring greater deformation to mobilize equivalent mechanical resistance.

(c) The experimental data demonstrate complex coupling effects among key parameters. The relationship between concrete strength and bond performance is modulated by cover thickness, following thick-walled cylinder theory principles. Enhanced concrete confinement from increased cover thickness shows diminishing returns beyond critical values, while longer bond lengths introduce stress distribution non-uniformities that reduce average bond strength.

(d) The bond-slip constitutive relationship exhibits distinct characteristics with increased rib spacing. The ascending branch shows reduced stiffness due to the enlarged concrete bearing zone, while maintaining similar peak bond stress. The post-peak behavior demonstrates more gradual degradation, particularly for specimens with adequate confinement. These modifications to the bond-slip response can be captured by adjusting key parameters in existing constitutive models, specifically the initial stiffness and softening coefficients, while preserving the fundamental mathematical framework.

## 7. Conclusions

A total of 42 pull-out specimens were designed and prepared to study the bond behavior between high-strength steel rebars with a yield strength of 630 MPa (T63) and concrete. The effects of crossrib spacing on the bond behavior between T63 rebar and concrete were analyzed in detail. Based on the results, the existing bond-slip constitutive model was applied to the rebar and the applicability of the existing constitutive model analyzed. From the above work, the following conclusions can be drawn:

(a) An increase of 30–50% in crossrib spacing had no significant effect on the bond strength between T63 rebar and concrete. The bond strength between two kinds of rebar (T63 and TB63) and concrete increased with increased concrete strength (*f*c) and relative protective layer thickness (c/d) and decreased with increased relative bond length (la/d).

(b) Crossrib spacing has no significant effect on the bond-slip curve between T63 rebar and concrete. Various test curves can be divided into the elastic stage, initial slip stage, peak stage, drop stage, and residual stage. The peak slippage of all kinds of specimens is small, at ~2 mm. When the crossrib spacing increased by 30–50%, the peak slippage between T63 rebar and concrete increased by nearly 50%.

(c) The calculation equation of the critical bond length of 630 MPa high-strength rebar was derived. The results showed that the critical bond length of this rebar could be calculated according to the Chinese Code (GB 50010-2010).

(d) The segmented bond-slip constitutive model was consistent with the test curve characteristics in this study and there was only a small difference in the slip characteristic points, which better described the bond-slip characteristics of 630 MPa high-strength rebar and concrete. Harajli’s constitutive model, which calculates the characteristic slip by the cross-ribs spacing, showed good applicability to rebar with different crossrib spacings.

(e) Based on these findings, it is recommended that the rib spacing limits for high-strength reinforcement could be appropriately relaxed while keeping other geometric parameters unchanged, as the modified spacing does not significantly affect bond capacity. The increased rib spacing can lead to reduced material consumption and simplified manufacturing processes, potentially resulting in cost savings while maintaining structural performance. When further optimizing the geometric design of high-strength reinforcement, both bond performance and manufacturing processes should be comprehensively considered, supporting potential modifications to current design codes and manufacturing standards.

## Figures and Tables

**Table 1:** Rebar crossrib spacing.

Crossrib Spacing Parameters	Rebar Diameter		
	16 mm	22 mm	25 mm
T63 crossrib spacing *l*_1_ (mm)	10	11	12.8
TB63 crossrib spacing *l*_2_ (mm)	13.8	14.5	19.8
(*l*_2_ − *l*_1_)/*l*_1_ (%)	38.0%	31.8%	54.7%

**Table 2:** Mechanical properties of rebar parameters.

Diameter *d* (mm)	Yield Strength *f*_y_ (N/mm^2^)	Ultimate Strength *f*_u_ (N/mm^2^)	*f*_u_/*f*_y_	Elongation After Break δ_h_	Elongation at Maximum Force δ_gt_	Elastic Modulus E (N/mm^2^)
16	633.2	818.7	1.29	21.9%	11.0%	2 × 10^5^
22	658.8	847.2	1.29	19.2%	10.3%	2 × 10^5^
25	655.0	856.0	1.31	18.5%	10.2%	2 × 10^5^

**Table 3:** Concrete ratio and mechanical properties.

Design Strength Grade	Mixing Ratio	Cube Compressive Strength (MPa)	Axial Compressive Strength (MPa)	Axial Tensile Strength (MPa)
	Cement:Fine Aggregates:Coarse Aggregates:Water			
C30	1:1.675:2.845:0.480	40.69	30.92	3.03
C40	1:1.703:2.898:0.400	50.32	38.24	3.41
C50	1:1.730:2.940:0.330	56.02	42.58	3.62

**Table 4:** Grouping of bonding specimens.

Specimen	Rebar	Diameter (mm)	Concrete Strength Grade	Bond Length (mm)
T6-5*d*-C30	T63	16	C30	80
T6-7*d*-C30	T63	16	C30	112
T6-10*d*-C30	T63	16	C30	160
T6-7*d*-C40	T63	16	C40	112
T6-7*d*-C50	T63	16	C50	112
T2-7*d*-C30	T63	22	C30	154
T5-7*d*-C30	T63	25	C30	175
TB6-5*d*-C30	TB63	16	C30	80
TB6-7*d*-C30	TB63	16	C30	112
TB6-10*d*-C30	TB63	16	C30	160
TB6-7*d*-C40	TB63	16	C40	112
TB6-7*d*-C50	TB63	16	C50	112
TB2-7*d*-C30	TB63	22	C30	154
TB5-7*d*-C30	TB63	25	C30	175

Note: T6 represents 630 MPa high-strength reinforcement (T63) with standard rib pattern; TB6 represents 630 MPa high-strength reinforcement (TB63) with increased rib spacing; The numbers 5*d*/7*d*/10*d* indicate bond lengths of 5, 7, and 10 times the bar diameter; C30/C40/C50 represent the design strength grades of concrete. For example, T6-7*d*-C30 denotes a specimen with standard T63 reinforcement, a bond length of 7*d*, and concrete strength grade C30.

**Table 5:** Failure modes of bonding specimens.

Specimen	Failure Mode	Specimen	Failure Mode
T6-5*d*-C30	Debonding	TB6-5*d*-C30	Debonding
T6-7*d*-C30	Debonding	TB6-7*d*-C30	Debonding
T6-10*d*-C30	Concrete splitting	TB6-10*d*-C30	Concrete splitting
T6-7*d*-C40	Concrete splitting	TB6-7*d*-C40	Debonding
T6-7*d*-C50	Concrete splitting	TB6-7*d*-C50	Concrete splitting
T2-7*d*-C30	Concrete splitting	TB2-7*d*-C30	Concrete splitting
T5-7*d*-C30	Concrete splitting	TB5-7*d*-C30	Concrete splitting

**Table 6:** Bond strength.

Specimen	Diameter of Rebar (mm)	Bond Length (mm)	Peak Load (kN)	Bond Strength (MPa)
T6-5*d*-C30	16	80	73.35	18.24 [0.55]
T6-7*d*-C30	16	112	82.25	14.61 [0.38]
T6-10*d*-C30	16	160	110.74	13.77 [1.22]
T6-7*d*-C40	16	112	119.91	21.30 [1.51]
T6-7*d*-C50	16	112	127.91	22.72 [1.48]
T2-7*d*-C30	22	154	108.14	10.16 [0.96]
T5-7*d*-C30	25	175	109.82	7.99 [0.77]
TB6-5*d*-C30	16	80	81.47	20.26 [1.86]
TB6-7*d*-C30	16	112	91.76	16.30 [0.81]
TB6-10*d*-C30	16	160	104.15	12.95 [1.65]
TB6-7*d*-C40	16	112	112.09	19.91 [0.89]
TB6-7*d*-C50	16	112	125.71	22.33 [1.43]
TB2-7*d*-C30	22	154	140.71	13.22 [1.12]
TB5-7*d*-C30	25	175	114.08	8.30 [0.93]

Note: “[ ]” standard deviation of bond strength.

**Table 7:** Characteristic points of the bond-slip curve.

Specimen	*τ*_u_ (MPa)	*S*_u_ (mm)	*τ*_r1_ (MPa)	*τ*_r2_ (MPa)	*τ*_r1_/*τ*_u_	*τ*_r2_/*τ*_u_
T6-5*d*-C30	18.24	1.67	8.8	7.01	48.25%	38.43%
T6-7*d*-C30	14.61	1.90	6.29	4.80	43.05%	32.85%
TB6-5*d*-C30	20.26	2.52	8.08	6.97	39.88%	34.40%
TB6-7*d*-C30	16.30	2.40	8.97	6.43	55.03%	39.45%
TB6-7*d*-C40	19.91	2.02	7.91	6.68	39.73%	33.55%

**Table 8:** Parameters of the bond-slip constitutive model in the Eurocode CEB-FIP Model Code.

Constraint Condition Failure Mode	Bonding State	Bonding Stress		Slippage		
		*τ*_u_	*τ*_r_/*τ*_u_	*S*_1_	*S*_2_	*S*_3_
Unconstrained concrete splitting failure	Good	$2\sqrt{{f_{c}}^{\prime }}$	0.15	0.6	0.6	1.0
	Fair	$\sqrt{{f_{c}}^{\prime }}$				2.5
Constrained debonding failure	Good	$2.5\sqrt{{f_{c}}^{\prime }}$	0.40	1.0	3.0	Cross-rib spacing
	Fair	$1.25\sqrt{{f_{c}}^{\prime }}$				

**Table 9:** Characteristic points of Xu’s bond-slip constitutive model.

Characteristic Points	Slip Point (*τ*_s_, *S*_s_)	Concrete Splitting Point (*τ*_cr_, *S*_cr_)	Ultimate Point (*τ*_u_, *S*_u_)	Residual Point (*τ*_r_, *S*_r_)
Bond strength *τ* MPa)	0.99*f*_t_	(1.6 + 0.7*c*/*d*)*f*_t_	(1.6 + 0.7*c*/*d* + 20*ρ*_sv_)*f*_t_	0.98*f*_t_
Slippage *s* (mm)	0.008*d*	0.0240*d*	0.0368*d*	0.540*d*

**Table 10:** Calculation parameters of Equation (18).

Parameter	Computing Formula	Note
*τ*_u_	$\sqrt{{f_{c}}^{\prime }}\frac{2.5}{1+3.1e^{-0.47K}}$	Ultimate bond strength
*B*	$\frac{0.0254+\rho_{\mathrm{sv}}}{-0.0232-8.34\rho_{\mathrm{sv}}}$	Descending section control parameters
*D*	$3\ln\left(\frac{0.7315+K}{5.176+0.3333K}-0.13\right)-3.375$	Rising section control parameters
*K*	$\frac{c}{d}+33\rho_{\mathrm{sv}}$	Constraint parameters

## Data Availability

The original contributions presented in this study are included in the article. Further inquiries can be directed to the corresponding author.

## Abbreviations

The following abbreviations are used in this manuscript:

T63	630 MPa high-strength steel rebar
TB63	T63 rebars with increased crossrib spacing
