# Correction: Chao, S. Influence of Crossrib Configuration on Bond-Slip Behavior for High-Strength Reinforcement in Concrete. *Materials* 2025, *18*, 3221

**DOI:** 10.3390/ma19132688

**Published:** 2026-06-23

**Authors:** Sisi Chao

**Affiliations:** 1 School of Architecture Engineering, Xi’an Technological University, No. 2 Xuefuzhonglu Road, Weiyang District, Xi’an 710021, China; chaosisi@xatu.edu.cn; Tel.: +86-18291820106; 2 Xi’an Key Laboratory of Urban Low-Carbon Construction, Chang’an University, Xi’an 710061, China

In the original publication [1], there was an error regarding the affiliation of Sisi Chao. Affiliation 2 “Xi’an Key Laboratory of Urban Low-Carbon Construction, Chang’an University, Xi’an 710061, China” was not included.

In the Funding Section, the sentence “Fundamental Research Funds for the Central Universities (No. 300102284501)“ should be updated to “Fundamental Research Funds for the Central Universities (CHD, Open Fund of Xi’an Key Laboratory of Urban Low-Carbon Construction, No. 300102284501)”. The authors state that the scientific conclusions are unaffected. This correction was approved by the Academic Editor. The original publication has also been updated.
